# Complete genome sequencing and evolutionary analysis of HCV subtype 6xg from IDUs in Yunnan, China

**DOI:** 10.1371/journal.pone.0217010

**Published:** 2019-05-16

**Authors:** Min Chen, Yanling Ma, Huichao Chen, Jie Dai, Hongbing Luo, Manhong Jia, Zhizhong Song

**Affiliations:** Institute for AIDS/STD Control and Prevention, Yunnan Center for Disease Control and Prevention, Kunming, Yunnan, China; Institut Pasteur of Shanghai Chinese Academy of Sciences, CHINA

## Abstract

**Background:**

HCV genotype 6 (HCV-6) typically circulates in Southeast Asia and exhibits the highest genetic diversity among the eight HCV genotypes. In our previous work, a group of HCV-6 sequences was not clearly classified. Here, we further characterized this HCV-6 variant and analyzed the evolutionary history of the enlarged HCV-6 family.

**Methods:**

Blood samples from eight HCV seropositive samples collected from intravenous drug users (IDUs) in 2014 in Yunnan Province, China. The full-length HCV genome sequences were amplified by using reverse transcription PCR followed by DNA sequencing and phylogenetic analysis. Bayesian evolutionary analysis was performed with the complete coding region sequences of subtype 6a-6xh.

**Results:**

The eight genomes had the same coding region of 9051 nucleotides. The complete coding region sequences of the eight HCV isolates formed a distinct phylogenetic group from the previously assigned HCV-6 subtypes (6a-6xf), however which clustered with 6xg reference sequences that were found in Kachin State, Myanmar, and recently assigned and released. The p-distances of the eight isolates to subtype 6a-6xf and 6xh ranged from 0.143 to 0.283. Based on the HCV-6 complete coding region sequences, we constructed a timescaled phylogenetic tree to reveal the HCV-6 evolutionary history, in which there were four HCV-6 phylogenetic subsets, whose median tMRCAs were 294.8, 388.5, 348.5 and 197.0 years ago, respectively. Subtype 6xg clustered into Subset I, and had the most recent common ancestor with subtype 6n, which dated back to 101.2 (95% HPD: 78.7, 125.8) years ago. The genetic evolutionary analysis further confirmed that subtype 6xg originated from Myanmar, and transmitted to Dehong through cross-border IDUs.

**Conclusion:**

The HCV-6 variant characterized in this study belonged to newly assigned subtype 6xg. Our finding further confirmed the assignment of 6xg. HCV-6 family was highly divers and had a complicated evolutionary history in Southeast Asia. It is necessary to further characterize HCV-6 genetics in this region.

## Introduction

Hepatitis C virus (HCV) belongs to the genus *Hepacivirus* in the family *Flaviviridae*. It has a single-stranded positive RNA genome of approximately 9600 nucleotides in length. The genome contains a single open reading frame (ORF) encoding a polyprotein, which further produce three structural (Core, E1, and E2) and seven non-structural proteins (p7, NS2, NS3, NS4A, NS4B, NS5A and NS5B). The untranslated regions (UTRs) flank the ORF in 5’ and 3’ terminal, which involves in protein translation and RNA replication. As a blood-borne pathogen, HCV infects an estimated 143 million people (2%) worldwide as of 2015 [1]. Persistent HCV infection is one of major causes of liver cirrhosis, hepatocellular carcinoma and liver failure [2].

The first full-length genome sequence of HCV was reported in 1989 [3], which promoted the development of HCV diagnosis and antiviral treatment [4]. However, HCV exhibits a high genetic diversity, the isolates from the different areas showed heterogeneity at the nucleotide level. Furthermore, these different isolates showed distinct origins, epidemiological history, even response to antiviral treatment [5, 6]. Thus, the classification of HCV is an important issue in HCV research. HCV classification is based on phylogenetic analyses of genome sequences, and include the genotype and subtype levels [7, 8]. Nowadays, HCV classified into eight genotypes, each of which contains a different number of subtypes. As of 2017, the number of confirmed subtypes increased to 86. Among the eight genotypes, genotype 6 predominantly distribute in South East Asia, where the most of subtypes from genotype 6 were identified [9].

Yunnan, a province in southwest China, borders Myanmar, Laos and Vietnam. Historically, Yunnan was a channel of heroin into China. Drug trafficking and intravenous drug use drove the development of HIV epidemic in Yunnan [10]. Stemmed from Yunnan, the HIV epidemic further spread to the other parts of China [11, 12]. Because intravenous drug use is also a major risk factor for HCV infection, co-infection with HCV and HIV-1 were common among IDUs in Yunnan. Recently, we carried out an HCV molecular epidemiological study among HIV-infected IDUs in Yunnan, which revealed the complicated and distinctive HCV genetics [13]. However, there was a group of HCV-6 sequences that were not clearly classified. In this study, we sequenced and characterized the full-length genome sequences of this group of isolates.

## Materials and methods

### Subjects and specimens

A total of eight HCV seropositive samples were collected from IDUs in Dehong Prefecture, China. The study subjects provided written informed consent. The study was approved by Biomedical Ethics Review Committee of Yunnan Center for Disease Control and Prevention.

### PCR amplification and sequencing

Plasma was separated from whole blood. Viral RNA was extracted from 140 μl of plasma by using the QIAamp Viral RNA Mini kit (Qiagen, Valencia, CA, United States) according to the manufacturer’s instructions. The cDNA was synthesized by using PrimeScript^TM^ 1^st^ Strand cDNA Synthesis Kit (Takara, Dalian, China). The target sequences were amplified in conventional nested or semi-nested PCRs. The PCR reaction was performed using TaKaRa LA Taq (Takara, Dalian, China). The overlapping PCR strategies and primers were shown in S1 Table. The PCR reaction programs were shown in S2 Table. The PCR products were sent to Qingke Co. (Kunming, China) for sequencing. The sequencing primers were shown in S3 Table.

### Sequence analysis

The original sequences were assembled with DNA sequence analysis software Sequencher 5.0 (Gene Codes, Ann Arbor, MI). The ClustalW Multiple alignment and manual editing were performed using Bio-Edit 7.0 software. The full-length sequences dataset consisted of the eight isolates from this study and the HCV reference sequences obtained from the website of International Committee on Taxonomy of Viruses (https://talk.ictvonline.org), covering HCV subtypes 6a-6xf. Phylogenetic tree analyses were performed using the maximum likelihood method based on the nucleotide substitution model GTR+I+G with 1000 bootstrap replicates, using MEGA (Molecular Evolutionary Genetics Analysis, version 6.0) [14]. Pairwise nucleotide similarities/distances were calculated as uncorrected p-distances using MEGA 6.0.

### Bayesian MCMC evolutionary analyses

Based on the complete coding region sequences, Bayesian Markov chain Monte Carlo (MCMC) method was used to construct a timescaled phylogenetic tree of HCV-6. According to the previous study, the uncorrelated lognormal relaxed molecular clock model is better than the exponential and strict clock models when analyzing the HCV complete coding region sequences [15]. Under the GTR+I+Γ4 nucleotide substitution model, Bayesian MCMC analyses were performed using the uncorrelated lognormal relaxed molecular clock model in combination with the Bayesian Skyline coalescent tree in the BEAST v1.8.2 package [16]. Because the dataset of HCV-6 complete coding region sequences lacked a sufficient temporal structure to estimate the evolutionary rate, an outer rate of 1.13×10^−3^ ±6.66×10^−6^ substitutions/site/year was used, which was used in a previous study [17]. Each MCMC analysis was run for 50 million generations and sampled every 5,000 generations. The MCMC analysis was repeated 12 times, the resulting log-files and tree-files were combined using LogCombiner v1.8.2, respectively. The combined log-file were analyzed in Tracer v1.5, all of the ESS members were >200. The Maximum Clade Credibility (MCC) tree was obtained from the combined tree-file by TreeAnnotator v1.7.4 with a burn-in of the initial 10% of generated trees, and examined by FigTree V1.3.1, which was also used to estimate the tMRCA of various nodes on the MCC tree.

### Sequence data

The nucleotide sequences obtained in this study were submitted to GenBank under the accession numbers MK139015-MK139022.

## Results

### Demographic characteristics of the study subjects

Recently, Wan et al found a potential new HCV-6 subtype among IDUs from the China-Myanmar border area by using four partial E1E2 (KM284973-KM284976) and five partial NS5B (KM285119-KM285123) sequences [18], which clustered with the eight sequences reported in one of our previous studies (Accession Numbers in Table 1) [13]. To clarify the classification of the eight strains, we further performed the complete genome sequencing and phylogenetic analysis for these strains. The eight samples were collected from eight HCV seropositive IDUs in Dehong Prefecture, bordering Myanmar. Among them, three were found in Yingjiang County, the other five were found in Longchuan County. One of them was Chinese, the other seven were Burmese. All of them were male, whose ages ranged from 19–33 years old (Table 1).

**Table 1:** The demographic characteristic of the eight isolates.

Sample ID	County	Nationality	Gender	Age	Accession Number for E1E2	Accession Number for NS5B	Accession Number for nearly full-length genome sequence
14DH34	Yingjiang	Chinese	Male	24	KT735417	KT735681	MK139015
14DH40	Longchuan	Burmese	Male	22	KT735422	KT735687	MK139016
14DH42	Longchuan	Burmese	Male	28	KT735424	KT735689	MK139017
14DH50	Yingjiang	Burmese	Male	29	KT735430	KT735697	MK139018
14DH51	Yingjiang	Burmese	Male	33	KT735431	KT735698	MK139019
14DH61	Longchuan	Burmese	Male	19	KT735438	KT735704	MK139020
14DH67	Longchuan	Burmese	Male	32	KT735442	KT735709	MK139021
14DH76	Longchuan	Burmese	Male	32	KT735444	KT735711	MK139022

### Genome sequences and organization

Nearly full-length genome sequences of HCV were obtained from the eight HCV isolates (14DH34, 14DH40, 14DH42, 14DH50, 14DH51, 14DH61, 14DH67 and 14DH76), which were 9451–9454 nucleotides in length, crossing from 5’UTR terminus to 3’UTR and covering the complete coding region of HCV, corresponding to the nucleotides numbered 1 to 9441–9444 in the H77 genome (S4 Table). The eight genomes had the same coding region of 9051 nt, in which the 10 protein-coding regions included: core (573 nt), E1 (576 nt), E2 (1092 nt), p7 (189 nt), NS2 (651 nt), NS3 (1893 nt), NS4A (162 nt), NS4B (783 nt), NS5A (1356 nt) and NS5B (1776 nt).

### Phylogenetic analysis of full-length genome sequences

**Fig 1:**
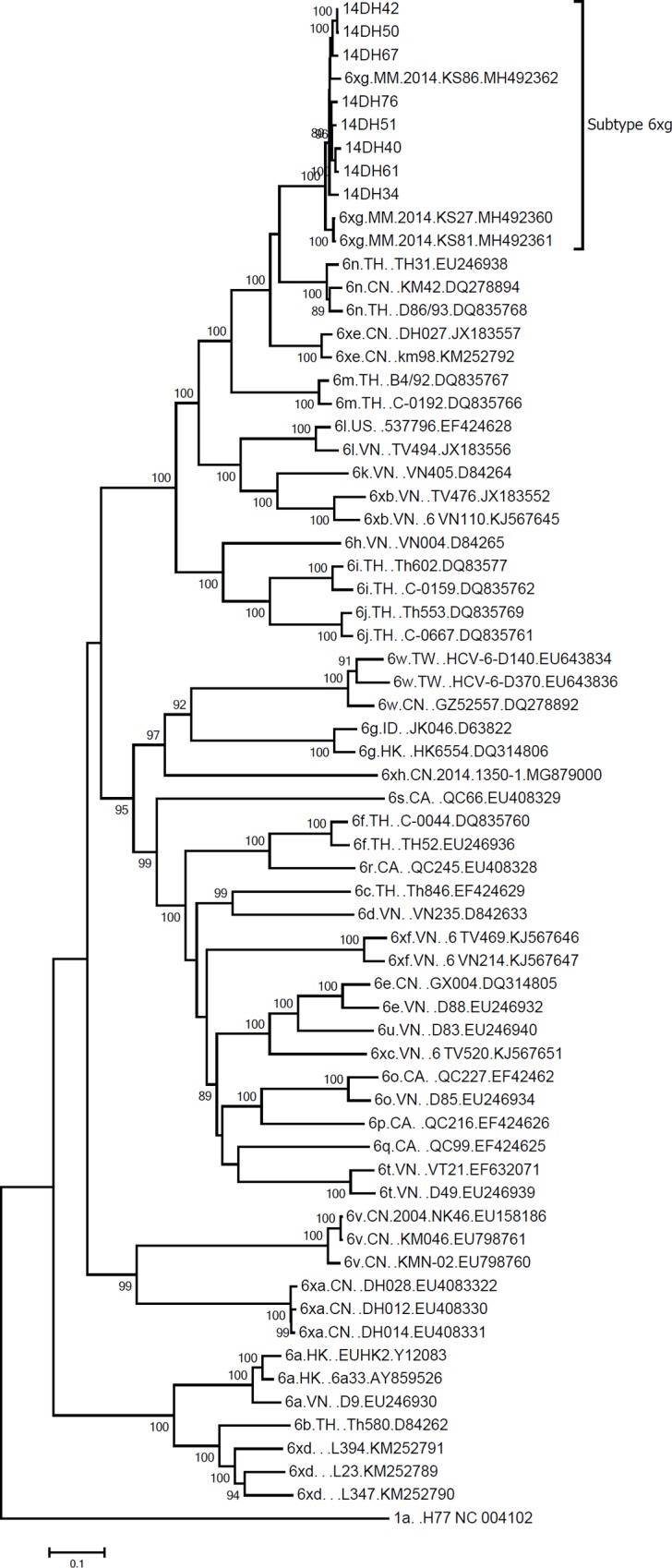
The maximum likelihood phylogenetic tree of HCV complete coding region sequences. The maximum likelihood phylogenetic tree was constructed with the complete coding region sequences of the eight isolates and the reference isolates of subtypes 6a-6xh. Values on the branches represent the percentage of 1000 bootstrap replicates. The scale bar indicates 1% nucleotide sequence divergence.

A maximum likelihood tree was constructed with the complete coding sequences of the eight isolates and 55 reference sequences of subtype 6a-6xf published on the website of International Committee on Taxonomy of Viruses (ICTV). The eight isolates formed a distinct phylogenetic clade with a strong bootstrap support, which was independent from the clades of subtype 6a-6xf and located between the clades of subtype 6n and 6xe. Then, we submitted the sequences to the ICTV Flaviviridae Study Group. After compared to the sequences in the database of the study group, the eight HCV sequences seemed to group with 6xg that was provisionally assigned in May 2018, however, whose reference sequences were not released. After the 6xg sequences (MH492360-MH492362) and 6xh sequence (MG879000) were released [19, 20], we repeated the analysis, which confirmed that the eight sequences were actually 6xg (Fig 1). Interestingly, the three 6xg sequences (MH492360-MH492362) were found in Kachin State, Myanmar [19]. Thus, the new HCV-6 subtype were reported in the three independent studies [13, 18, 19], which suggested that there were multiple distinct epidemiological unrelated isolates.

### Pairwise comparison of nucleotide sequences

Based on the complete coding region, the pairwise distances of subtype 6a-6xh to these eight isolates were calculated, which ranged from 0.028 to 0.283 (Table 2). Except for subtype 6xg, subtype 6n and subtype 6xe showed less different from the eight isolates, the p-distances were 0.143–0.146 and 0.146–0.151. A genetic distance threshold to separate isolates of the same subtype from those of different subtypes is usually 13–15% [7].

**Table 2:** The pairwise distances of subtype 6a-6xf to the eight isolates.

	14DH34	14DH40	14DH42	14DH50	14DH51	14DH61	14DH67	14DH76	Mean
6a_Y12083	0.279	0.283	0.282	0.283	0.281	0.283	0.281	0.279	0.281
6b_D84262	0.271	0.275	0.275	0.275	0.272	0.275	0.273	0.273	0.274
6c_EF424629	0.257	0.260	0.260	0.260	0.258	0.260	0.261	0.258	0.259
6d_D84263	0.259	0.264	0.262	0.263	0.261	0.263	0.263	0.262	0.262
6e_DQ314805	0.266	0.267	0.267	0.268	0.265	0.268	0.268	0.266	0.267
6f_DQ835760	0.263	0.265	0.264	0.265	0.263	0.263	0.265	0.263	0.264
6g_D63822	0.272	0.271	0.273	0.274	0.271	0.271	0.272	0.272	0.272
6h_D84265	0.233	0.235	0.235	0.235	0.231	0.234	0.236	0.234	0.234
6i_DQ835770	0.232	0.231	0.233	0.234	0.229	0.231	0.233	0.231	0.232
6j_DQ835769	0.232	0.232	0.235	0.235	0.231	0.232	0.234	0.232	0.233
6k_D84264	0.220	0.220	0.223	0.223	0.218	0.219	0.221	0.217	0.220
6l_EF424628	0.212	0.214	0.215	0.215	0.211	0.212	0.214	0.212	0.213
6m_DQ835767	0.185	0.188	0.187	0.188	0.187	0.188	0.187	0.187	0.187
**6n_DQ835768**	**0.144**	**0.146**	**0.145**	**0.145**	**0.143**	**0.145**	**0.144**	**0.143**	**0.144**
6o_EF424627	0.258	0.260	0.261	0.262	0.259	0.260	0.259	0.258	0.260
6p_EF424626	0.258	0.258	0.259	0.260	0.256	0.256	0.260	0.257	0.258
6q_EF424625	0.250	0.251	0.252	0.252	0.249	0.252	0.252	0.251	0.251
6r_EU408328	0.258	0.262	0.262	0.262	0.259	0.262	0.262	0.260	0.261
6s_EU408329	0.271	0.274	0.273	0.273	0.272	0.273	0.274	0.270	0.272
6t_EF632071	0.260	0.263	0.264	0.264	0.261	0.263	0.263	0.263	0.263
6u_EU246940	0.263	0.268	0.268	0.268	0.266	0.266	0.266	0.265	0.266
6v_EU158186	0.266	0.268	0.271	0.271	0.268	0.268	0.272	0.268	0.269
6w_DQ278892	0.269	0.271	0.272	0.272	0.269	0.269	0.272	0.269	0.270
6xa_EU408330	0.250	0.254	0.254	0.254	0.252	0.255	0.255	0.252	0.253
6xb_JX183552	0.222	0.222	0.225	0.225	0.220	0.220	0.224	0.221	0.222
6xc_KJ567651	0.262	0.266	0.266	0.267	0.262	0.265	0.266	0.263	0.265
6xd_KM252789	0.268	0.269	0.271	0.272	0.269	0.270	0.271	0.269	0.270
**6xe_JX183557**	**0.148**	**0.149**	**0.150**	**0.151**	**0.147**	**0.148**	**0.148**	**0.146**	**0.148**
6xf_KJ567646	0.266	0.268	0.268	0.268	0.266	0.266	0.269	0.266	0.267
6xg_MH492360	0.036	0.035	0.034	0.035	0.028	0.032	0.036	0.031	0.033
6xh_MG879000	0.265	0.266	0.267	0.267	0.266	0.266	0.267	0.266	0.266

#### Genetic evolutionary history of HCV-6

With the continuous discovery of new HCV-6 subtypes, the taxonomic structure of genotype 6 continued to change. To better understand the genetic history of HCV-6, we constructed a timescaled phylogenetic tree with the complete coding sequences of subtypes 6a-6xh. After searching the HCV database (https://hcv.lanl.gov) and the website of International Committee on Taxonomy of Viruses (https://talk.ictvonline.org), there were just 90 HCV-6 nearly full-length genome sequences, among which just nine had the annotation of collection date (S5 Table). Because of the lack of a sufficient temporal structure to estimate an evolutionary rate, we had to use an outer rate as the prior when performing Bayesian MCMC analysis, which was applied in the previous work [17, 21–25].

**Fig 2:**
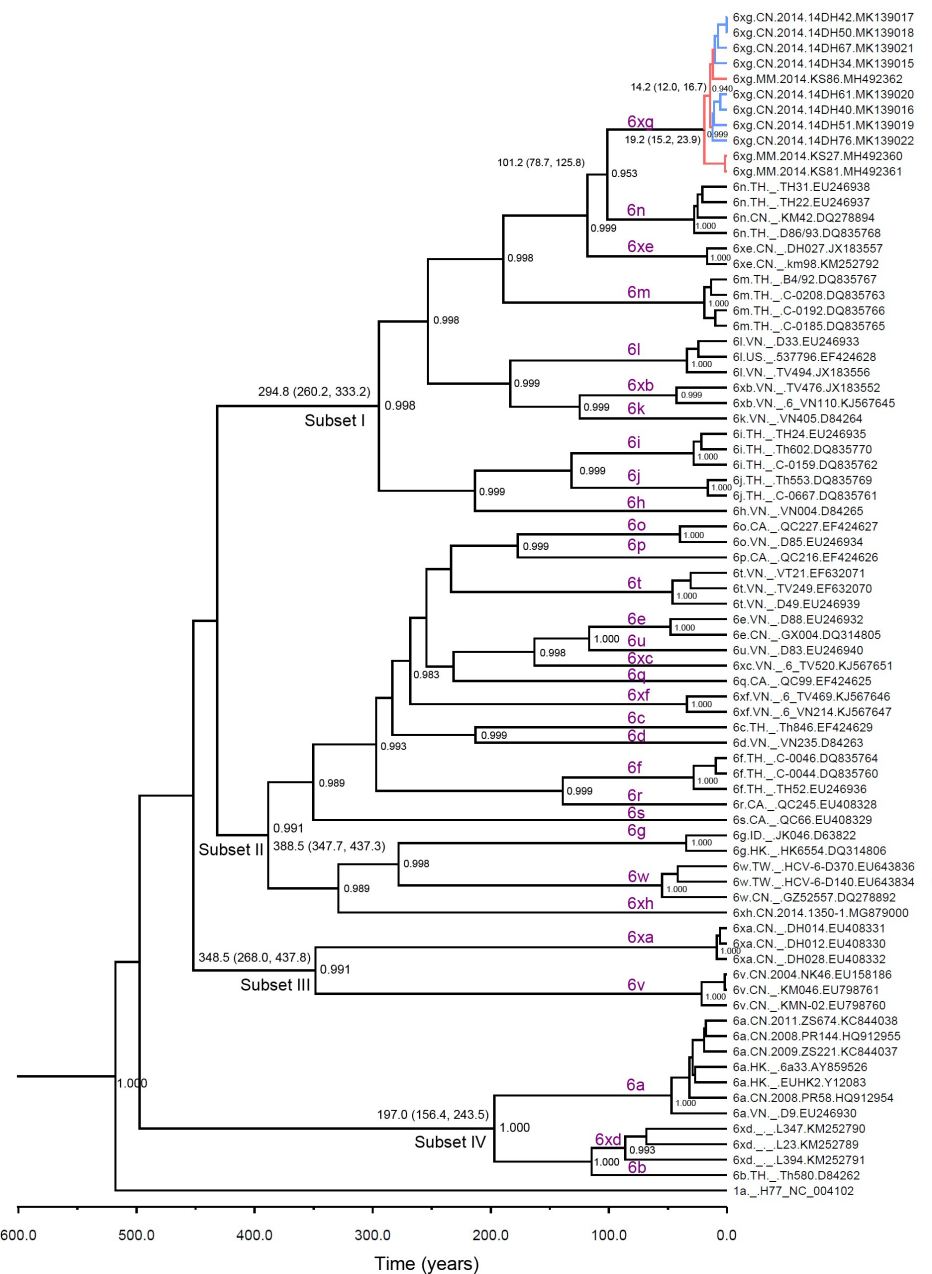
Maximum clade credibility (MCC) tree representing the rooted genealogy of HCV-6 subtypes. The branch lengths in the MCC trees reflect time and the corresponding time-scale is shown at the bottom of the trees. The posterior probabilities greater than 0.9 were shown at the respective nodes. The tMRCA for the interested nodes are shown as median (95% HPD).

The maximum clade credibility (MCC) tree of genotype 6 showed that subtypes 6a-6xh were divided into four phylogenetic subsets (Fig 2). The median time of most recent common ancestor (tMRCA) of Subset I dated back to 294.8 (95% highest posterior density (HPD): 260.2, 333.2) years ago, including 6h, 6i, 6j, 6k, 6l, 6m, 6n, 6xb, 6xe and 6xg. The most of HCV-6 subtypes belonged to Subset II, whose median tMRCA dated back to 388.5 (95% HPD: 347.7, 437.3) years ago, including 6c, 6d, 6e, 6f, 6g, 6o, 6p, 6q, 6r, 6s, 6t, 6u, 6w, 6xc, 6xf and 6xh. The median tMRCA of Subset III dated back to 348.5 (95% HPD: 268.0, 437.8), just including 6v and 6xa. Subset IV was the youngest, whose median tMRCA dated back to 197.0 (95% HPD: 156.4, 243.5) years ago, including 6a, 6b and 6xd.

As shown in Fig 2, subtype 6xg clustered into Subset I, and had the most recent common ancestor with subtype 6n, which dated back to 101.2 (95% HPD: 78.7, 125.8) years ago. In the clade of subtype 6xg, the isolates from Myanmar (MH492360 and MH492361) were direct descendants of the most recent 6xg common ancestor dated back to 19.2 years ago. Branched from the isolates from Myanmar, a clade including isolates from Dehong and Myanmar descended from a common ancestor dated back to 14.2 years ago. These suggested that 6xg originated in Myanmar and transmitted into Dehong.

## Discussion

In 2005, the consensus proposal for nomenclature of HCV genotypes and subtypes was issued, which confirmed the assignment of six genotypes and 18 subtypes [8]. Because the lack of a complete coding region sequence or additional isolates, the other 58 subtypes were provisionally assigned. Based on the consensus proposal, to confirm whether an HCV isolate is a new genotype or subtype, at least one complete coding region sequence should be obtained, and the followed criteria should be met: 1) Form a distinct phylogenetic clade from previously confirmed sequences; b) At least three epidemiologically unlinked isolates are identified; c) Does not represent a recombinant between other genotypes or subtypes. After that, 38 provisional subtypes were confirmed [7]. However, the proposal has been complicated by the increasing volume of HCV sequences. In 2014, the genotypes and subtypes assignments and the nomenclature rules were updated [7], however, the criteria for assigning genotypes and subtypes remain unchanged. The changes included: a provisional subtype is not assigned without a complete genome sequence; how to assign the subtypes numbering beyond “w”; and how to name the intergenotypic recombinant forms. Meanwhile, the ICTV Flaviviridae Study Group is responsible to take a coordinating role in the assignment of newly described variants of HCV. To June 2017, the number of confirmed subtypes increased to 86.

The eight HIV-6 isolates characterized in this study were collected in 2014 in Dehong, Yunnan, China, whose partial E1E2 and NS5B sequences were firstly misclassified as subtype 6n [13]. Soon after, Wan et al found that four E1E2 and five NS5B sequences from IDUs in 2011–2012 could be a potential new HCV-6 subtype, interestingly, which clustered with the counterparts of the eight isolates reported in our previous study [18]. Because of the lack of the complete genome sequences, the potential new HCV-6 subtype could not be assigned. Thus, we further characterized the full-length genomes of the eight HCV-6 isolates collected in 2014. The complete genome sequences of the eight isolates formed an independent clade in phylogenetic tree, and cluster with 6xg (MH492360-MH492362) that was provisionally assigned in May 2018 and released in November 13 2018. The three 6xg sequences (MH492360-MH492362) were found in Kachin State, Myanmar by Ye et al [19]. So far, the new HCV-6 subtype were found in three independent studies [13, 18, 19], which suggested that there were multiple distinct epidemiological unrelated isolates. The present study further confirmed the assignment of 6xg.

Among the eight HCV genotypes, genotype 6 is more diverse than the other seven genotypes, which suggests that HCV-6 is to represent an oldest HCV lineage [26–29]. The number of confirmed HCV-6 subtypes had increased from six in 2005 to 29 as of June 2017, accounting for one-third of all confirmed subtypes [7, 8]. However, the number of HCV-6 complete genome sequences was limited, there were just 90 HCV-6 nearly full-length genome sequences which could be found in the HCV database (https://hcv.lanl.gov) and the website of International Committee on Taxonomy of Viruses (https://talk.ictvonline.org). Among them, there were just nine with the annotation of collection date (S5 Table), which is important to construct the temporal structure when performing evolutionary analysis [15, 30]. Although the partial gene sequences were also used for evolutionary analysis, the complete coding region sequences could maximize the phylogenetic resolution [15]. Thus, the more nearly full-length genome sequences with history archives are necessary to reveal HCV evolutionary history. The lack of a sufficient temporal structure to estimate an evolutionary rate is a long-standing problem in the field of HCV research [15, 17, 21–25, 31]. The previous studies used an outer evolutionary rate as the prior to perform Bayesian MCMC analysis, which opens the door to investigate the epidemic history of HCV [15, 17, 21–25, 31]. The evolutionary rate used in this study was also utilized to analyze multiple HCV subtypes in the previous studies [17]. By this way, for the first time, we preliminarily constructed the evolutionary history with the complete coding region sequences of the HCV-6 family. A previous study showed that the approximate tMRCA of the complete coding region sequences from HCV-6 subfamily was 300–400 years ago [17], which coincided with the timescale found in our study. In future, to improve the time resolution, it is necessary to exactly estimate the evolutionary rate with the more nearly full-length genome sequences well annotated. So far, subtype 6xg nearly full-length genome sequences were annotated best among HCV-6 subtypes. We believe that ICTV Flaviviridae Study Group would get this work forward.

Southeast Asia is the ancestral and endemic region of HCV-6. The most of HCV-6 subtypes were identified in this region, including Vietnam, Thailand, Laos, Myanmar and Yunnan Province in China, where HCV-6 showed very high variability [13, 22, 27, 28, 32–36]. In this study, we found that there were four phylogenetic subsets of HCV-6 subtypes circulating in Southeast Asia. Each phylogenetic subset contained HCV-6 strains coming from the different countries, which suggested that HCV-6 had a complicated evolutionary history in this area, and at least some historical gene flow occurred. Furthermore, some newly identified HCV-6 subtypes had already circulated, such as 6xd, whose median tMRCA dated back to 86.1 years ago. Otherwise, this region contains a proportion of novel and unclassified HCV-6 variants. HCV subtype has particular epidemiological implications. As genetic markers, HCV subtypes are used to trace the transmission and evolution of HCV in specific population or areas. Thus, it is necessary to further characterize HCV-6 subtypes in this region.

According to Hepatitis C Guidance 2018 Update [37], for treatment-naïve patients with Genotype 5 and Genotype 6 infection, three regimens are recommended, namely glecaprevir/pibrentasvir, sofosbuvir/velpatasvir and ledipasvir/sofosbuvir. To date, there is no special regimen for a certain HCV-6 subtype. However, the nearly full-length genome sequences of novel HCV-6 subtypes provide the basic for the research about the interaction between HCV and direct-acting antiviral agents (DAAs), such as naturally occurring drug resistance.

The previous studies showed that HIV-1 subtypes B and C were first introduced into IDUs in Dehong which borders Myanmar [38, 39], and further generated a number of BC recombinant forms [40]. Our previous study also showed that HCV subtype 1a, 6n and 6u among IDUs transmitted from Dehong to the other part of Yunnan, which suggested that Dehong was an entry for these subtypes [13]. In this study, the subtype 6xg was mostly identified among Burmese IDUs staying in Dehong, which suggested that this variant could come from Myanmar. The genetic evolutionary analysis further confirmed that subtype 6xg originated from Myanmar, and transmitted to Dehong through cross-border IDUs. With the development of economics, a certain amount of Burmese commuted across the China-Myanmar border, which increased the possibility of the introduction of pathogen [41, 42]. The blood-borne diseases prevention and control in border areas should be further concerned.

In this study, we characterized the nearly full-length genome sequences of subtype 6xg collected, which confirmed the circulation of subtype 6xg in China, and supported the assignment of 6xg. The genetic evolutionary analysis depicted the whole picture of the enlarged HCV-family, and improved our knowledge about the evolutionary histories of HCV-6 subtypes.

## Supporting information

S1 Table PCR primers. (PDF)

S2 Table PCR programs. (PDF)

S3 Table Sequencing primers. (PDF)

S4 Table The number of nucleotides in each genomic region ORF. (PDF)

S5 Table The Nearly full-length genome sequences of HCV-6 subtypes. (PDF)
