# Supplementary material for: Complete genome sequencing and evolutionary analysis of HCV subtype 6xg from IDUs in Yunnan, China
Source: PLoS One. 2019 May 16;14(5):e0217010. doi: 10.1371/journal.pone.0217010 (PMC6522032; doi:10.1371/journal.pone.0217010)
Supplement: S1 Table — (PDF) [file pone.0217010.s001.pdf]

S1 Table. PCR Primers

Segment	Nested PCR	Primer name	Direction	Primer sequence (5'-3')	Position	Size
1	1st PCR	5UTRF	Forward	GCCAGCCCCTAAYGGGGCGA	1-20	404
		5UTR1R	Reverse	ACTCCACCAACGATCTGACCGCC	442-420	
	2nd PCR	5UTRF	Forward	GCCAGCCCCTAAYGGGGCGA	1-20	
		5UTR1R	Reverse	GTCCTGTGGGCGACGGTTGGT	404-348	
2	1st PCR	A1F	Forward	TGTCTTCACGCAGAAAGCGTCTA	59-81	1136
		A1R	Reverse	TCATCATCATATCCCACGCCATTCT	1314-1290	
	2nd PCR	A2F	Forward	CCATGGCGTTAGTATGAGTGTCGT	83-106	
		A2R	Reverse	AGGTGAAAAGCTGTCCTACCAAGAA	1218-1194	
3	1st PCR	C/E2-1F	Forward	GCCGACCTCATGGGRTAYAT	732-751	1304
		C/E2-1R	Reverse	ARTTBTYDGTRCANGGRTARTGCCA	2211-2187	
	2nd PCR	C/E2-2F	Forward	CCYGGTTGCTCYTTYTCTATCTT	849-871	
		C/E2-2R	Reverse	GTNADCCARGGHCCNGMNCCRCA	2152-2130	
4	1st PCR	B1F	Forward	CCTTCAGCTGCCTACGTCGTT	1323-1343	2363
		B1R	Reverse	CTCCCGAGGACCCTTTGAGAGT	3840-3819	
	2nd PCR	B2F	Forward	TGAGGATACCGCAATTGCTCCTC	1354-1376	
		B2R	Reverse	ACAAGCGCACGGTGTTAGTGA	3716-3696	
5	1st PCR	NS3-1F	Forward	ATGGARAAGAARRTYATYRTITGGG	3276-3300	650
		NS3-2F	Forward	ATGGARAYYAAGVTYATYACITGGG		
		NS3-1R	Reverse	CTYTTICCRCTICCIGTIGGIGCRTG	4051-4026	
	2nd PCR	nestNS3-1F	Forward	tgtaaaacgacggccagtATCTTICTIGGICCIG CYGA	3369-3388	
		nestNS3-2F	Forward	tgtaaaacgacggccagtATACTICTIGGICCIG CIGA		
		nestNS3-R	Reverse	caggaaacagctatgaccGCIACYTGRTAIGTI TGIGG	4018-3999	
6	1st PCR	C1F	Forward	CGGTTTACCATGGTGCTGGCT	3580-3600	2971
		C1R	Reverse	TTCGCACGACCTCGACGTACT	6627-6607	
	2nd PCR	C2F	Forward	AAGGCCCTGTGTGCCAGATGTA	3622-3643	
		C2R	Reverse	CTCCAAAGAGCCCTCTCATAGTTGG	6592-6568	
7	1st PCR	D1F	Forward	TGCACCGTGCTGTCTGACTTC	6294-6314	2791
		D1R	Reverse	TCTCACAGCCCAGTTGAAGAGGT	9194-9172	
	2nd PCR	D2F	Forward	GGCTCAAGGCAAAGCTTGTACC	6322-6343	
		D2R	Reverse	GCTCGATGTCTCCAAGCTCTCAAT	9112-9089	
8	1st PCR	NS5B-1F	Forward	GGSTTYTCGTATGAYACCMGBTGYT TTGA	8247-8275	1038
		NS5B-1R	Reverse	CTACCCCTACRGSRAGYAGGAGTAG GC	9351-9325	
	2nd PCR	NS5B-2F	Forward	GCTGYTTTGAYTCAACNGTCAC	8266-8287	
		NS5B-2R	Reverse	GRGCMYGRGACACGCTGTGATASAT GTC	9303-9276	
9	1st PCR	E1F	Forward	AATCACTCCACTTGATCTCCCAG	8960-8982	386

	E1R	Reverse	AAGCAGTGGTATCAACGCAGAGAAA<br>AAAAAAAAAAAAAAAAAAAAAA	9442-9418
2nd PCR	E2F	Forward	GGAGCATGCCTCAGAAAACTTG	9057-9078
	E2R	Reverse	AAGCAGTGGTATCAACGCAGAG	9442-9418
