# Supplementary material for: Complete genome sequencing and evolutionary analysis of HCV subtype 6xg from IDUs in Yunnan, China
Source: PLoS One. 2019 May 16;14(5):e0217010. doi: 10.1371/journal.pone.0217010 (PMC6522032; doi:10.1371/journal.pone.0217010)
Supplement: S2 Table — (PDF) [file pone.0217010.s002.pdf]

S2 Table. PCR programs

Segment	Nested PCR	PCR Programs
1	1st PCR	94◦C for 3 min, followed by 35 cycles with 94◦C for 30 sec, 62◦C for 35 sec, and 72◦C for 45 sec, then 72◦C for 10 min.
	2nd PCR	94◦C for 3 min, followed by 35 cycles with 94◦C for 30 sec, 62◦C for 35 sec, and 72◦C for 45 sec, then 72◦C for 10 min.
2	1st PCR	94◦C for 3 min, followed by 35 cycles with 94◦C for 30 sec, 58◦C for 35 sec, and 72◦C for 1 min 20 sec, then 72◦C for 10 min.
	2nd PCR	94◦C for 3 min, followed by 35 cycles with 94◦C for 30 sec, 58◦C for 35 sec, and 72◦C for 1 min 20 sec, then 72◦C for 10 min.
3	1st PCR	94◦C for 1 min, followed by 3 cycles with 94◦C for 1 min, 50◦C for 1 min, and 72◦C for 2 min, then 32 cycles with 94◦C for 15 sec, 50◦C for 30 sec, and 72◦C for 1min 30 sec, then 72◦C for 10 min.
	2nd PCR	94◦C for 1 min, followed by 3 cycles with 94◦C for 1 min, 55◦C for 1 min, and 72◦C for 2 min, then 32 cycles with 94◦C for 15 sec, 55◦C for 30 sec, and 72◦C for 1min 20 sec, then 72◦C for 10 min.
4	1st PCR	94◦C for 3 min, followed by 35 cycles with 94◦C for 30 sec, 58◦C for 35 sec, and 72◦C for 2 min 30 sec, then 72◦C for 10 min.
	2nd PCR	94◦C for 3 min, followed by 35 cycles with 94◦C for 30 sec, 58◦C for 35 sec, and 72◦C for 2 min 30 sec, then 72◦C for 10 min.
5	1st PCR	94◦C for 3 min, followed by 35 cycles of 94◦C for 15 s, 53◦C for 1 min, and 72◦C for 1 min, then 72◦C for 10 min.
	2nd PCR	94◦C for 5 min, followed by 5 cycles with 94◦C for 30 sec, 53◦C for 1 min, and 72◦C for 1 min, then 30 cycles with 94◦C for 15 sec, 67◦C for 30 sec, and 72◦C for 30 sec, then 72◦C for 10 min.
6	1st PCR	94◦C for 3 min, followed by 35 cycles with 94◦C for 30 sec, 58◦C for 35 sec, and 72◦C for 3 min, then 72◦C for 10 min.
	2nd PCR	94◦C for 3 min, followed by 35 cycles with 94◦C for 30 sec, 58◦C for 35 sec, and 72◦C for 3 min, then 72◦C for 10 min.
7	1st PCR	94◦C for 3 min, followed by 35 cycles with 94◦C for 30 sec, 58◦C for 35 sec, and 72◦C for 3 min, then 72◦C for 10 min.
	2nd PCR	94◦C for 3 min, followed by 35 cycles with 94◦C for 30 sec, 58◦C for 35 sec, and 72◦C for 3 min, then 72◦C for 10 min.
8	1st PCR	94◦C for 1 min, followed by 3 cycles with 94◦C for 1 min, 50◦C for 1 min, and 72◦C for 2 min, then 32 cycles with 94◦C for 15 sec, 50◦C for 30 sec, and 72◦C for 1min 30 sec, then 72◦C for 10 min.
	2nd PCR	94◦C for 1 min, followed by 3 cycles with 94◦C for 1 min, 55◦C for 1 min, and 72◦C for 2 min, then 32 cycles with 94◦C for 15 sec, 55◦C for 30 sec, and 72◦C for 1min 20 sec, then 72◦C for 10 min.
9	1st PCR	94◦C for 3 min, followed by 35 cycles with 94◦C for 30 sec, 56◦C for 35 sec, and 72◦C for 45 sec, then 72◦C for 10 min.
	2nd PCR	94◦C for 3 min, followed by 35 cycles with 94◦C for 30 sec, 56◦C for 35 sec, and 72◦C for 45 sec, then 72◦C for 10 min.
