# Supplementary material for: Complete genome sequencing and evolutionary analysis of HCV subtype 6xg from IDUs in Yunnan, China
Source: PLoS One. 2019 May 16;14(5):e0217010. doi: 10.1371/journal.pone.0217010 (PMC6522032; doi:10.1371/journal.pone.0217010)
Supplement: S3 Table — (PDF) [file pone.0217010.s003.pdf]

S3 Table. Sequencing primers

Segment	Primer name	Direction	Primer sequence (5'-3')	Position
1	5UTRF	Forward	GCCAGCCCCTAAYGGGGCGA	1-20
	5UTR1R	Reverse	GTCCTGTGGGCGACGGTTGGT	404-348
2	A2F	Forward	CCATGGCGTTAGTATGAGTGTCGT	83-106
	A2R	Reverse	AGGTGAAAAGCTGTCCTACCAAGAA	1218-1194
3	C/E2-2F	Forward	CCYGGTTGCTCYTTYTCTATCTT	849-871
	C/E2-2R	Reverse	GTNADCCARGGHCCNGMNCCRCA	2152-2130
4	B2F	Forward	TGAGGATACCGCAATTGCTCCTC	1354-1376
	B2R	Reverse	ACAAGCGCACGGTGTTAGTGA	3716-3696
	BSF1	Forward	ATCCGGAGACGACATACGCAAA	2107-2128
	BSR1	Reverse	TTGGAACAAGTAAAGAGGGCCAAG	3050-3027
5	M13F	Forward	TGTAAAACGACGGCCAGT	3369-3388
	M13R	Reverse	CAGGAAACAGCTATGACC	4018-3999
6	C2F	Forward	AAGGCCCTGTGTGCCAGATGTA	3622-3643
	C2R	Reverse	CTCCAAAGAGCCCTCTCATAGTTGG	6592-6568
	CSF1	Forward	TGGATCGATCACCGTCCCACA	4397-4417
	CSR1	Reverse	ACTTACCACAAAAGCCGTAGAGGC	5837-5814
	CSF2	Forward	GGGCGATTACTTATTCTACCTATGG	4027-4231
	CSR2	Reverse	TCTACGCAGTAGACTGGTTATGGTG	6218-6194
7	D2F	Forward	GGCTCAAGGCAAAGCTTGTACC	6322-6343
	D2R	Reverse	GCTCGATGTCTCCAAGCTCTCAAT	9112-9089
	DSF1	Forward	TCATCCCCGAGTATGATGACAGAG	7117-7139
	DSR1	Reverse	CAGTATAGCCTCTCAGTCAAAGATG	8386-8362
	DSF2	Forward	GGACGTCATGGTCGTTACATCAAT	6839-6862
	DSF3	Forward	AAAGGATGTCCGGAGTCATGCTAG	7916-7938
8	NS5B-2F	Forward	GCTGYTTTGAYTCAACNGTCAC	8266-8287
	NS5B-2R	Reverse	GRGCMYGRGACACGCTGTGATASATGTC	9303-9276
9	E2F	Forward	GGAGCATGCCTCAGAAAACTTG	9057-9078
	E2R	Reverse	AAGCAGTGGTATCAACGCAGAG	9442-9418
