# Supplementary material for: Complete genome sequencing and evolutionary analysis of HCV subtype 6xg from IDUs in Yunnan, China
Source: PLoS One. 2019 May 16;14(5):e0217010. doi: 10.1371/journal.pone.0217010 (PMC6522032; doi:10.1371/journal.pone.0217010)
Supplement: S4 Table — (PDF) [file pone.0217010.s004.pdf]

S4 Table. The number of nucleotides in each genomic region ORF

Subtype	Isolated	Length	ORF	5'UTR	Core	E1	E2	p7	NS2	NS3	NS4A	NS4B	NS5A	NS5B	3'UTR
1a	H77	9646	9036	341	573	576	1089	189	651	1893	162	783	1344	1776	269
6xg	14DH34	9453	9051	338	573	576	1092	189	651	1893	162	783	1356	1776	64
6xg	14DH40	9453	9051	338	573	576	1092	189	651	1893	162	783	1356	1776	64
6xg	14DH42	9451	9051	338	573	576	1092	189	651	1893	162	783	1356	1776	62
6xg	14DH50	9452	9051	338	573	576	1092	189	651	1893	162	783	1356	1776	63
6xg	14DH51	9453	9051	338	573	576	1092	189	651	1893	162	783	1356	1776	64
6xg	14DH61	9453	9051	338	573	576	1092	189	651	1893	162	783	1356	1776	64
6xg	14DH67	9454	9051	338	573	576	1092	189	651	1893	162	783	1356	1776	65
6xg	14DH76	9453	9051	338	573	576	1092	189	651	1893	162	783	1356	1776	64
