# Supplementary material for: Complete genome sequencing and evolutionary analysis of HCV subtype 6xg from IDUs in Yunnan, China
Source: PLoS One. 2019 May 16;14(5):e0217010. doi: 10.1371/journal.pone.0217010 (PMC6522032; doi:10.1371/journal.pone.0217010)
Supplement: S5 Table — (PDF) [file pone.0217010.s005.pdf]

S5 Table. The Nearly full-length sequences of HCV-6 subtypes

	Name	Collection date	Submitted	Reference
1	6a.HK._.6a72.DQ480522	N/A	05-APR-2006	Direct Submission
2	6a.HK._.6a65.DQ480518	N/A	05-APR-2006	Direct Submission
3	6a.HK._.6a73.DQ480517	N/A	05-APR-2006	Direct Submission
4	6a.HK._.6a61.DQ480516	N/A	05-APR-2006	Direct Submission
5	6a.HK._.6a64.DQ480515	N/A	05-APR-2006	Direct Submission
6	6a.HK._.6a63.DQ480514	N/A	05-APR-2006	Direct Submission
7	6a.HK._.6a35.DQ480513	N/A	05-APR-2006	Direct Submission
8	6a.HK._.6a77.DQ480512	N/A	05-APR-2006	Direct Submission
9	6a.HK._.6a74.DQ480524	N/A	05-APR-2006	Direct Submission
10	6a.HK._.6a62.DQ480523	N/A	05-APR-2006	Direct Submission
11	6a.HK._.6a69.DQ480521	N/A	05-APR-2006	Direct Submission
12	6a.HK._.6a67.DQ480520	N/A	05-APR-2006	Direct Submission
13	6a.HK._.6a66.DQ480519	N/A	05-APR-2006	Direct Submission
14	6a.CN.2008.PR144.HQ912955	2008	20-JAN-2011	Direct Submission
15	6a.CN.2008.PR58.HQ912954	2008	20-JAN-2011	Direct Submission
16	6a.CN._.WYHCV2.HQ639936	N/A	20-NOV-2010	Direct Submission
17	6a.CN.2011.ZS674.KC844038	2011	27-MAR-2013	[1]
18	6a.CN.2009.ZS221.KC844037	2009	27-MAR-2013	[1]
19	6a.HK._.EUHK2.Y12083	N/A	25-Mar-1997	[2, 3]
20	6a.HK._.6a33.AY859526	N/A	17-Dec-2004	Direct Submission
21	6a.VN._.D9.EU246930	N/A	24-Oct-2007	[4, 5]
22	6b.TH._.Th580.D84262	N/A	29-Mar-1996	[6, 7]
23	6c.TH._.Th846.EF424629	N/A	06-FEB-2007	[7, 8]
24	6d.VN._.VN235.D84263	N/A	29-MAR-1996	[6, 9, 10]
25	6e.CN._.GX004.DQ314805	N/A	02-DEC-2005	[11, 12]
26	6e.VN._.D88.EU246932	N/A	24-OCT-2007	[4, 5]
27	6e.US._.537798.EU408326	N/A	16-JAN-2008	Direct Submission
28	6e.VN._.D42.EU246931	N/A	24-OCT-2007	[4, 5]
29	6f.TH._.C-0046.DQ835764	N/A	23-JUN-2006	[13, 14]
30	6f.TH._.C-0044.DQ835760	N/A	23-JUN-2006	[13, 14]
31	6f.TH._.TH52.EU246936	N/A	24-OCT-2007	[4, 5]
32	6g.ID._.JK046.D63822	N/A	10-AUG-1995	[15]
33	6g.HK._.HK6554.DQ314806	N/A	02-DEC-2005	[11, 12]
34	6h.VN._.VN004.D84265	N/A	29-MAR-1996	[6, 9, 10]
35	6i.TH._.Th602.DQ835770	N/A	23-JUN-2006	[7, 13, 16]
36	6i.TH._.C-0159.DQ835762	N/A	23-JUN-2006	[13, 14]
37	6i.TH._.TH24.EU246935	N/A	24-OCT-2007	[4, 5]
38	6j.TH._.Th553.DQ835769	N/A	23-JUN-2006	[7, 13, 16]
39	6j.TH._.C-0667.DQ835761	N/A	23-JUN-2006	[13, 14]
40	6k.CN._.KM41.DQ278893	N/A	03-NOV-2005	[17, 18]
41	6k.CN._.KM45.DQ278891	N/A	03-NOV-2005	[17, 18]
42	6k.VN._.VN405.D84264	N/A	29-MAR-1996	[6, 9, 10]

43	6l.US._.537796.EF424628	N/A	06-FEB-2007	[8, 19]
44	6l.VN._.D33.EU246933	N/A	24-OCT-2007	[4, 5]
45	6l.VN._.TV317.JX183555	N/A	15-JUN-2012	[20, 21]
46	6l.VN._.TV494.JX183556	N/A	15-JUN-2012	[20, 21]
47	6m.TH._.B4/92.DQ835767	N/A	23-JUN-2006	[13, 22, 23]
48	6m.TH._.C-0192.DQ835766	N/A	23-JUN-2006	[13, 14]
49	6m.TH._.C-0185.DQ835765	N/A	23-JUN-2006	[13, 14]
50	6m.TH._.C-0208.DQ835763	N/A	23-JUN-2006	[13, 14]
51	6n.CN._.KM42.DQ278894	N/A	06-JAN-2005	[17]
52	6n.TH._.D86/93.DQ835768	N/A	23-JUN-2006	[13, 22, 23]
53	6n.TH._.TH31.EU246938	N/A	24-OCT-2007	[4, 5]
54	6n.TH._.TH22.EU246937	N/A	24-OCT-2007	[4, 5]
55	6o.CA._.QC227.EF424627	N/A	06-FEB-2007	[8]
56	6o.US._.535318.EU408327	N/A	16-JAN-2008	Direct Submission
57	6o.VN._.D85.EU246934	N/A	24-OCT-2007	[4, 5]
58	6p.CA._.QC216.EF424626	N/A	06-FEB-2007	[8]
59	6q.CA._.QC99.EF424625	N/A	06-FEB-2007	[8]
60	6r.CA._.QC245.EU408328	N/A	16-JAN-2008	[24, 25]
61	6s.CA._.QC66.EU408329	N/A	16-JAN-2008	[24, 25]
62	6t.VN._.VT21.EF632071	N/A	24-MAY-2007	[26]
63	6t.VN._.TV249.EF632070	N/A	24-MAY-2007	[26]
64	6t.VN._.TV241.EF632069	N/A	24-MAY-2007	[26]
65	6t.VN._.D49.EU246939	N/A	24-OCT-2007	[4, 5]
66	6u.VN._.D83.EU246940	N/A	24-OCT-2007	[4, 5]
67	6v.CN.2004.NK46.EU158186	2004	16-SEP-2007	[26]
68	6v.CN._.KMN-02.EU798760	N/A	06-JUN-2008	[27]
69	6v.CN._.KM181.FJ435090	N/A	06-JUN-2008	[27]
70	6v.CN._.KM046.EU798761	N/A	06-JUN-2008	[27]
71	6w.CN._.GZ52557.DQ278892	N/A	03-NOV-2005	[11, 17]
72	6w.TW._.HCV-6-D140.EU643834	N/A	15-APR-2008	[28]
73	6w.TW._.HCV-6-D370.EU643836	N/A	15-APR-2008	[28]
74	6xa.CN._.DH012.EU408330	N/A	16-JAN-2008	[29, 30]
75	6xa.CN._.DH028.EU408332	N/A	16-JAN-2008	[29, 30]
76	6xa.CN._.DH014.EU408331	N/A	16-JAN-2008	[29, 30]
77	6xb.VN._.TV476.JX183552	N/A	15-JUN-2012	[20, 21]
78	6xb.VN._.6_VN110.KJ567645	N/A	11-MAR-2014	[31, 32]
79	6xc.VN._.6_TV520.KJ567651	N/A	11-MAR-2014	[31, 32]
80	6xd._._.L23.KM252789	N/A	31-JUL-2014	[33]
81	6xd._._.L347.KM252790	N/A	31-JUL-2014	[33]
82	6xd._._.L394.KM252791	N/A	31-JUL-2014	[33]
83	6xe.CN._.DH027.JX183557	N/A	15-JUN-2012	[20, 30]
84	6xe.CN._.km98.KM252792	N/A	31-JUL-2014	[33, 34]
85	6xf.VN._.6_TV469.KJ567646	N/A	11-MAR-2014	[31, 32]
86	6xf.VN._.6_VN214.KJ567647	N/A	11-MAR-2014	[31, 32]

87	6xg.MM.2014.KS27.MH492360	2014	14-JUN-2018	[35]
88	6xg.MM.2014.KS81.MH492361	2014	14-JUN-2018	[35]
89	6xg.MM.2014.KS86.MH492362	2014	14-JUN-2018	[35]
90	6xh.CN.2014.1350-1.MG879000	2014	13-NOV-2018	[36]
91	6xg.CN.2014.14DH34.MK139015	2014	04-NOV-2018	
92	6xg.CN.2014.14DH40.MK139016	2014	04-NOV-2018	
93	6xg.CN.2014.14DH42.MK139017	2014	04-NOV-2018	
94	6xg.CN.2014.14DH50.MK139018	2014	04-NOV-2018	
95	6xg.CN.2014.14DH51.MK139019	2014	04-NOV-2018	
96	6xg.CN.2014.14DH61.MK139020	2014	04-NOV-2018	
97	6xg.CN.2014.14DH67.MK139021	2014	04-NOV-2018	
98	6xg.CN.2014.14DH76.MK139022	2014	04-NOV-2018	

References

1. Xu R, Tong W, Gu L, Li C, Fu Y, Lu L. A panel of 16 full-length HCV genomes was characterized in China belonging to genotypes 1-6 including subtype 2f and two novel genotype 6 variants. Infect Genet Evol. 2013;20:225-9. Epub 2013/09/10. https://doi.org/10.1016/j.meegid.2013.08.014 PMID: 24012950.
2. Adams NJ, Chamberlain RW, Taylor LA, Davidson F, Lin CK, Elliott RM, et al. Complete coding sequence of hepatitis C virus genotype 6a. Biochem Biophys Res Commun. 1997;234(2):393-6. Epub 1997/05/19. https://doi.org/10.1006/bbrc.1997.6627 PMID: 9177282.
3. Simmonds P, McOmish F, Yap PL, Chan SW, Lin CK, Dusheiko G, et al. Sequence variability in the 5' non-coding region of hepatitis C virus: identification of a new virus type and restrictions on sequence diversity. J Gen Virol. 1993;74 (Pt 4):661-8. Epub 1993/04/01. https://doi.org/10.1099/0022-1317-74-4-661 PMID: 8385694.
4. Noppornpanth S, Poovorawan Y, Lien TX, Smits SL, Osterhaus AD, Haagmans BL. Complete genome analysis of hepatitis C virus subtypes 6t and 6u. J Gen Virol. 2008;89(Pt 5):1276-81. Epub 2008/04/19. https://doi.org/10.1099/vir.0.83593-0 PMID: 18420806.
5. Noppornpanth S, Sablon E, De Nys K, Truong XL, Brouwer J, Van Brussel M, et al. Genotyping hepatitis C viruses from Southeast Asia by a novel line probe assay that simultaneously detects core and 5' untranslated regions. J Clin Microbiol. 2006;44(11):3969-74. Epub 2006/09/08. https://doi.org/10.1128/JCM.01122-06 PMID: 16957039.
6. Tokita H, Okamoto H, Iizuka H, Kishimoto J, Tsuda F, Miyakawa Y, et al. The entire nucleotide sequences of three hepatitis C virus isolates in genetic groups 7-9 and comparison with those in the other eight genetic groups. J Gen Virol. 1998;79 (Pt 8):1847-57. Epub 1998/08/26. https://doi.org/10.1099/0022-1317-79-8-1847 PMID: 9714232.
7. Tokita H, Okamoto H, Luengrojanakul P, Vareesangthip K, Chainuvati T, Iizuka H, et al. Hepatitis C virus variants from Thailand classifiable into five novel genotypes in the sixth (6b), seventh (7c, 7d) and ninth (9b, 9c) major genetic groups. J Gen Virol. 1995;76 (Pt 9):2329-35. Epub 1995/09/01. https://doi.org/10.1099/0022-1317-76-9-2329 PMID: 7561773.
8. Lu L, Li C, Fu Y, Gao F, Pybus OG, Abe K, et al. Complete genomes of hepatitis C virus (HCV) subtypes 6c, 6l, 6o, 6p and 6q: completion of a full panel of genomes for HCV genotype 6. J Gen Virol. 2007;88(Pt 5):1519-25. Epub 2007/04/07. https://doi.org/10.1099/vir.0.82820-0 PMID: 17412981.

9. Tokita H, Okamoto H, Tsuda F, Song P, Nakata S, Chosa T, et al. Hepatitis C virus variants from Vietnam are classifiable into the seventh, eighth, and ninth major genetic groups. Proc Natl Acad Sci U S A. 1994;91(23):11022-6. Epub 1994/11/08. PMID: 7972001.
10. Song P, Duc DD, Hien B, Nakata S, Chosa T, Watanabe J, et al. Markers of hepatitis C and B virus infections among blood donors in Ho Chi Minh City and Hanoi, Vietnam. Clin Diagn Lab Immunol. 1994;1(4):413-8. Epub 1994/07/01. PMID: 8556478.
11. Li C, Fu Y, Lu L, Ji W, Yu J, Hagedorn CH, et al. Complete genomic sequences for hepatitis C virus subtypes 6e and 6g isolated from Chinese patients with injection drug use and HIV-1 co-infection. J Med Virol. 2006;78(8):1061-9. Epub 2006/06/22. https://doi.org/10.1002/jmv.20663 PMID: 16789024.
12. Zhang L, Chen Z, Cao Y, Yu J, Li G, Yu W, et al. Molecular characterization of human immunodeficiency virus type 1 and hepatitis C virus in paid blood donors and injection drug users in china. J Virol. 2004;78(24):13591-9. Epub 2004/11/27. https://doi.org/10.1128/JVI.78.24.13591-13599.2004 PMID: 15564470.
13. Lu L, Li C, Fu Y, Thaikruea L, Thongswat S, Maneekarn N, et al. Complete genomes for hepatitis C virus subtypes 6f, 6i, 6j and 6m: viral genetic diversity among Thai blood donors and infected spouses. J Gen Virol. 2007;88(Pt 5):1505-18. Epub 2007/04/07. https://doi.org/10.1099/vir.0.82604-0 PMID: 17412980.
14. Thaikruea L, Thongsawat S, Maneekarn N, Netski D, Thomas DL, Nelson KE. Risk factors for hepatitis C virus infection among blood donors in northern Thailand. Transfusion. 2004;44(10):1433-40. Epub 2004/09/24. https://doi.org/10.1111/j.1537-2995.2004.04073.x PMID: 15383015.
15. Tokita H, Okamoto H, Iizuka H, Kishimoto J, Tsuda F, Lesmana LA, et al. Hepatitis C virus variants from Jakarta, Indonesia classifiable into novel genotypes in the second (2e and 2f), tenth (10a) and eleventh (11a) genetic groups. J Gen Virol. 1996;77 (Pt 2):293-301. https://doi.org/10.1099/0022-1317-77-2-293 PMID: 8627233.
16. Luengrojanakul P, Vareesangthip K, Chainuvati T, Murata K, Tsuda F, Tokita H, et al. Hepatitis C virus infection in patients with chronic liver disease or chronic renal failure and blood donors in Thailand. J Med Virol. 1994;44(3):287-92. Epub 1994/11/01. PMID: 7531758.
17. Lu L, Nakano T, He Y, Fu Y, Hagedorn CH, Robertson BH. Hepatitis C virus genotype distribution in China: predominance of closely related subtype 1b isolates and existence of new genotype 6 variants. J Med Virol. 2005;75(4):538-49. https://doi.org/10.1002/jmv.20307 PMID: 15714489.
18. Lu L, Nakano T, Li C, Fu Y, Miller S, Kuiken C, et al. Hepatitis C virus complete genome sequences identified from China representing subtypes 6k and 6n and a novel, as yet unassigned subtype within genotype 6. J Gen Virol. 2006;87(Pt 3):629-34. https://doi.org/10.1099/vir.0.81400-0 PMID: 16476984.
19. Nainan OV, Alter MJ, Kruszon-Moran D, Gao FX, Xia G, McQuillan G, et al. Hepatitis C virus genotypes and viral concentrations in participants of a general population survey in the United States. Gastroenterology. 2006;131(2):478-84. Epub 2006/08/08. https://doi.org/10.1053/j.gastro.2006.06.007 PMID: 16890602.
20. Wang H, Yuan Z, Barnes E, Yuan M, Li C, Fu Y, et al. Eight novel hepatitis C virus genomes reveal the changing taxonomic structure of genotype 6. J Gen Virol. 2013;94(Pt 1):76-80. Epub 2012/09/28. https://doi.org/10.1099/vir.0.047506-0 PMID: 23015745.

21. Pham VH, Nguyen HD, Ho PT, Banh DV, Pham HL, Pham PH, et al. Very high prevalence of hepatitis C virus genotype 6 variants in southern Vietnam: large-scale survey based on sequence determination. Jpn J Infect Dis. 2011;64(6):537-9. PMID: 22116339.
22. Apichartpiyakul C, Chittivudikarn C, Miyajima H, Homma M, Hotta H. Analysis of hepatitis C virus isolates among healthy blood donors and drug addicts in Chiang Mai, Thailand. J Clin Microbiol. 1994;32(9):2276-9. Epub 1994/09/01. PMID: 7814558.
23. Doi H, Apichartpiyakul C, Ohba KI, Mizokami M, Hotta H. Hepatitis C virus (HCV) subtype prevalence in Chiang Mai, Thailand, and identification of novel subtypes of HCV major type 6. J Clin Microbiol. 1996;34(3):569-74. Epub 1996/03/01. PMID: 8904416.
24. Li C, Lu L, Zhang X, Murphy D. Entire genome sequences of two new HCV subtypes, 6r and 6s, and characterization of unique HVR1 variation patterns within genotype 6. J Viral Hepat. 2009;16(6):406-17. https://doi.org/10.1111/j.1365-2893.2009.01086.x PMID: 19281488.
25. Murphy DG, Willems B, Deschenes M, Hilzenrat N, Mousseau R, Sabbah S. Use of sequence analysis of the NS5B region for routine genotyping of hepatitis C virus with reference to C/E1 and 5' untranslated region sequences. J Clin Microbiol. 2007;45(4):1102-12. https://doi.org/10.1128/JCM.02366-06 PMID: 17287328.
26. Lu L, Murphy D, Li C, Liu S, Xia X, Pham PH, et al. Complete genomes of three subtype 6t isolates and analysis of many novel hepatitis C virus variants within genotype 6. J Gen Virol. 2008;89(Pt 2):444-52. https://doi.org/10.1099/vir.0.83460-0 PMID: 18198375.
27. Wang Y, Xia X, Li C, Maneekarn N, Xia W, Zhao W, et al. A new HCV genotype 6 subtype designated 6v was confirmed with three complete genome sequences. J Clin Virol. 2009;44(3):195-9. https://doi.org/10.1016/j.jcv.2008.12.009 PMID: 19179105.
28. Lee YM, Lin HJ, Chen YJ, Lee CM, Wang SF, Chang KY, et al. Molecular epidemiology of HCV genotypes among injection drug users in Taiwan: Full-length sequences of two new subtype 6w strains and a recombinant form_2b6w. J Med Virol. 2010;82(1):57-68. https://doi.org/10.1002/jmv.21658 PMID: 19950240.
29. Xia X, Zhao W, Tee KK, Feng Y, Takebe Y, Li Q, et al. Complete genome sequencing and phylogenetic analysis of HCV isolates from China reveals a new subtype, designated 6u. J Med Virol. 2008;80(10):1740-6. https://doi.org/10.1002/jmv.21287 PMID: 18712831.
30. Xia X, Lu L, Tee KK, Zhao W, Wu J, Yu J, et al. The unique HCV genotype distribution and the discovery of a novel subtype 6u among IDUs co-infected with HIV-1 in Yunnan, China. J Med Virol. 2008;80(7):1142-52. https://doi.org/10.1002/jmv.21204 PMID: 18461611.
31. Li C, Pham VH, Abe K, Lu L. Nine additional complete genome sequences of HCV genotype 6 from Vietnam including new subtypes 6xb and 6xc. Virology. 2014;468-470:172-7. Epub 2014/09/01. https://doi.org/10.1016/j.virol.2014.08.006 PMID: 25173194.
32. Li C, Yuan M, Lu L, Lu T, Xia W, Pham VH, et al. The genetic diversity and evolutionary history of hepatitis C virus in Vietnam. Virology. 2014;468-470:197-206. https://doi.org/10.1016/j.virol.2014.07.026 PMID: 25193655.
33. Li C, Barnes E, Newton PN, Fu Y, Vongsouvath M, Klenerman P, et al. An expanded taxonomy of hepatitis C virus genotype 6: Characterization of 22 new full-length viral genomes. Virology. 2015;476:355-63. https://doi.org/10.1016/j.virol.2014.12.025 PMID: 25589238.
34. Lu L, Wang M, Xia W, Tian L, Xu R, Li C, et al. Migration patterns of hepatitis C virus in China characterized for five major subtypes based on samples from 411 volunteer blood donors from 17 provinces and municipalities. J Virol. 2014;88(13):7120-9. https://doi.org/10.1128/JVI.00414-14

PMID: 24719413.
35. Ye M, Chen X, Wang Y, Duo L, Zhang C, Zheng Y-T. Identification of a new HCV subtype 6xg among injection drug users in Kachin, Myanmar. Frontiers in Microbiology. 2019;10:814. https://doi.org/10.3389/fmicb.2019.00814.
36. Wu T, Xing Z, Yuan M, Ge J, Yuan G, Liang K, et al. Analysis of HCV Isolates Among the Li Ethnic in Hainan Island of South China Reveals Their HCV-6 Unique Evolution and a New Subtype. Cell Physiol Biochem. 2018;50(5):1832-9. Epub 2018/11/06. https://doi.org/10.1159/000494863 PMID: 30396187.
